# Immediately modifiable risk factors attributable to colorectal cancer in Malaysia

**DOI:** 10.1186/s12889-017-4650-8

**Published:** 2017-08-04

**Authors:** Cho Naing, Pei Kuan Lai, Joon Wah Mak

**Affiliations:** 1 School of Postgraduate Studies, International Medical University, 57000 Kuala Lumpur, Malaysia; 2 College of Public Health, Medical and Veterinary Sciences, James Cook University, Townsville, 4811 Queensland Australia

**Keywords:** Colorectal cancer, Population-attributable fraction, Risk factors, Malaysia

## Abstract

**Background:**

This study aimed to estimate potential reductions in case incidence of colorectal cancer attributable to the modifiable risk factors such as alcohol consumption, overweight and physical inactivity amongst the Malaysian population.

**Methods:**

Gender specific population-attributable fractions (PAFs) for colorectal cancer in Malaysia were estimated for the three selected risk factors (physical inactivity, overweight, and alcohol consumptions). Exposure prevalence were sourced from a large-scale national representative survey. Risk estimates of the relationship between the exposure of interest and colorectal cancer were obtained from published meta-analyses. The overall PAF was then estimated, using the 2013 national cancer incidence data from the Malaysian Cancer Registry.

**Results:**

Overall, the mean incidence rate for colorectal cancer in Malaysia from 2008 to 2013 was 21.3 per 100,000 population, with the mean age of 61.6 years (±12.7) and the majority were men (56.6%). Amongst 369 colorectal cancer cases in 2013, 40 cases (20 men, 20 women), 10 cases (9 men, 1 woman) or 20 cases (16 men,4 women) would be prevented, if they had done physical exercises, could reduce their body weight to normal level or avoided alcohol consumption, assuming that these factors are causally related to colorectal cancer. It was estimated that 66 (17.8%;66/369) colorectal cancer cases (42 men, 24 women) who had all these three risk factors for the last 10 years would have been prevented, if they could control these three risk factors through effective preventive measures.

**Conclusions:**

Findings suggest that approximately 18% of colorectal cancer cases in Malaysia would be prevented through appropriate preventive measures such as doing regular physical exercises, reducing their body weight to normal level and avoiding alcohol consumption, if these factors are causally related to colorectal cancer. Scaling-up nationwide public health campaigns tailored to increase physical activity, controlling body weight within normal limits and avoid alcohol intake are recommended. Future studies with other site-specific cancers and additional risk factors are needed.

## Background

Due to the global demographic and epidemiologic transitions, evidence for an increasing cancer burden over the next decades has emerged, particularly in low and middle income countries [1, 2]. The International Agency for Research of Cancer (IARC) reported in 2012 that lung, breast, and colorectal cancers (CRCs) were the most commonly diagnosed cancers, while lung, liver, and stomach cancers were the most common causes of cancer death [3]. Globally, CRC was the third most common form of cancer [3, 4]. In Malaysia, according to the second report of the National Cancer Patient Registry (NCPR-CC), CRC was the second most common cancer after breast cancer [5–7].

Public health strategies that focus on reducing cancer incidences can benefit from comparing risk factors which have potential population-level impact [8]. Behavioural risk factors, for example physical inactivity, alcohol consumption and smoking are amenable to modification. The IARC has classified as “sufficient evidence” on linking consumption of alcohol beverage [9], physical inactivity and overweight to cancers, including colon, rectum, and female breast cancer [10, 11].

Implementing modifications in any of these (undesirable) behaviours would require considerable effort in health education and relevant health interventions. To justify such preventive strategies, it is important to quantify the theoretical impacts of changes on site-specific cancer incidence at the population level [12]. Prevalence of risk factors among Malaysian population is available in the National Health and Morbidity Surveys, including the National Health and Morbidity Survey 2006 (NHMS III 2006) [13, 14]. The population-level impact of individual risk factors and their combinations depend on both the relative risk (RR) and the risk factor distribution (i.e. exposure prevalence) in the target population [15]. As such, an epidemiologic metric, the population attributable fraction (PAF), which is the fraction of all cases (exposed and unexposed) that would not have occurred if exposure had not occurred [15, 16]. In essence, PAF takes into consideration both disease incidence and risk factor prevalence. Such calculation requires an estimate of strength of association (i.e. RR) between a given risk factor (e.g. physical inactivity) and a particular cancer (CRC in this case) and the prevalence of the risk factor in the population [16, 17]. By virtue of the availability of nationally representative data such as the cancer registry (i.e. NCPR-CC) and t nationally representative population-based surveys, it is possible to do estimations of PAF among the Malaysia population in relation to three selected modifiable factors (alcohol consumption, overweight/obesity and physical inactivity), which could be attributed to increase the rate of CRC.

On the whole, the objective of the present study was to estimate potential reductions in case incidence of CRC attributable to the selected modifiable risk factors (alcohol consumption, overweight and physical inactivity) amongst the Malaysian population.

## Methods

### Colorectal cancer incidence data

CRC incidence data for the year 2013 was sourced from the 2nd NCPR-CC, which is a nationwide cancer patient registry for CRC for the period 2008–2013. The registration of multiple primary cancers is in accordance with the IARC criteria [6, 7]. Details of data collection process are available elsewhere [5].

### Prevalence of risk factors

The selection of risk factor was based on (i) the evidence of their associations with CRC, (ii) the potentially preventable factors (modifiable factors), and (iii) the availability of data. Information on the evidences of potential risk factors associated with CRC were obtained from the IARC report [10, 11]. We obtained exposure prevalence from the NHMS III 2006. Details about this national survey are described elsewhere [13, 18]. In brief, the NHMS III 2006 was conducted in 2006, which was designed to provide data at both national and state level about population living in private households in Malaysia. It used a two-stage stratified random sampling proportionately to the population size for selection of the enumeration blocks and the living quarters (LQs). A total of 33,933 household heads of the selected LQs were interviewed using questionnaire which the answers represent the information for the members of the household [13]. Physical inactivity was defined as having a total physical activity level of less than 600 metabolic equivalents-minutes per week (METs-minutes*/*week) contributed by all three different life domains (work, travelling and leisure time) [13, 19, 20]. Alcohol consumption in the NHMS IIII 2006 survey was regarded as a current drinker, which was defined as those who still consumed alcohol for the past 1 month prior to the survey [13]. Obesity was defined as BMI 35–39.9 kg/mm^2^, while overweight was BMI 25–29.9 kg/mm^2^ and normal weight was BMI 18.5–24.9 kg/mm^2^ [13].

Assuming a conservative latent period of 10 years between exposure/consumption of risk factors and diagnosis of CRC (i.e. the lag period for CRC development), it was better to use prevalence of exposure from population-based survey earlier than 2003 for the CRC cases in 2013. We could obtain data on exposure prevalence of overweight/obesity, alcohol consumption and physical inactivity only from the NHMS III 2006 data [13]. We, therefore, assumed that the trends of these risk factors among Malaysian population were likely stable between 2003 and 2006. Sources of exposure prevalence data and its key characteristics were presented in Table 1.

**Table 1:** Parameter values and the estimated population attributable fraction for colorectal cancer in Malaysia, 2013

Risk factor	Description	Prevalence% (95% CI)^a^	Relative risk (95% CI)	meta-analysis study for relative risk	Colorectal cancer cases	PAF% (95% CI)	Preventable cases (95% CI)
Alcohol (current drinkers, ≥18 years)	Men	13.7 (12.6–14.7)	1.62 (1.31–2.21)	[22]	201	7.8 (3.8–12.9)	16 (8–26)
	Women	0.4 (0.035–0.5)	1.54 (1.04–2.29)	[22]	168	2.1 (0–6)	4 (0–9)
	By ethnicity						
	Malay	0.8 (0.04–0.1)	1.52 (1.21–1.81)	[22]	174	0.4 (0.01–0.8)	0 (0–1)
	Chinese	26.6 (24.5–28.5)	1.52 (1.21–1.81)	[22]	134	12.2 (4.9–8.0)	16 (7–25)
	Indian	14.9 (12.8–17.8)	1.52 (1.21–1.81)	[22]	22	7.2 (2.6–18.8)	2 (1–3)
	Others	7.7 (4.7–10.8)	1.52 (1.21–1.81)	[22]	35	3.8 (2.2–18.8)	2 (1–3)
Physical inactivity (an inverse of physical activity)	Men	35.3 (34.3–36.3)	1.32 (1.22–1.41)	[23]	201	10.1 (7–13)	20 (14–26)
	Women	50.5 (49.5–51.5)	1.26 (1.13–1.41)	[23]	168	11.6 (6–17.4)	20 (10–29)
	By ethnicity						
	Malay	42.4 (41.3–43.4)	1.28 (1.16–1.42)	[23]	174	10.6 (6.2–15.4)	18 (11–27)
	Chinese	47.1 (45.6–48.7)	1.28 (1.16–1.42)	[23]	134	11.7 (6.8–17)	16 (9–23)
	Indian	44.5 (42.2–46.7)	1.28 (1.16–1.42)	[23]	22	11.7 (6.3–16.4)	2 (1–4)
	Others	41.3 (38.2–44.4)	1.28 (1.16–1.42)	[23]	35	10.4 (5.8–15.8)	4 (1–5)
Overweight	Men	29.7 (28.9–30.5)	1.16 (1.07–1.27)	[21]	201	4.5 (2–7.6)	9 (4–15)
	Women	28.6 (27.9–29.3)	1.03 (0.96–1.1)	[21]	168	0.9 (0–2.8)	1 (0–5)
	By ethnicity						
	Malay	29.8 (28.5–35.1)	1.09 (1.02–1.15)	[21]	174	4.5 (2–7.6)	5 (1–6)
	Chinese	28.5 (27.3–29.6)	1.09 (1.02–1.15)	[21]	134	2.5 (0.5–4.3)	3 (1–6)
	Indian	33.2 (31.4–35.1)	1.09 (1.02–1.15)	[21]	22	2.9 (0.4–5)	1 (0–1)
	Others	20.8 (18.8–23)	1.09 (1.02–1.15)	[21]	35	2.9 (0.4–5)	1 (0–1)

^a^Exposure prevalence obtained from the national health and morbidity survey 2006 [14]; PAF: population attributable fraction; women including missing data

### Estimates of relative risk

Estimates of the relationship between exposures of interest and CRC were obtained from the most recently available high quality meta-analyses [21–24].

### Statistical analysis

#### Estimation based on a single risk factor

PAF (proportion of CRC occurring in the Malaysian population that could be attributed to individual risk factor) was calculated, using a formula as follows [16]:

$$ PAF=\left[\mathrm{P}\left(RR-1\right)\right]/\left[\left[\mathrm{P}\ \mathrm{x}\ \left(RR-1\right)\right]+1\right] $$

Where,

P = prevalence of exposure to the risk factor in total population;

RR = relative risk of risk factor and a specific cancer (CRC in this case).

#### Estimation based on multiple risk factors

Combined PAF for exposure to two risk factors *A* and *B* can be calculated by the following formula [8]:

$$ {PAF}_{\mathrm{A}\mathrm{B}}=1-\left(1-{PAF}_{\mathrm{A}}\right)\ \mathrm{x}\ \left(1-{PAF}_{\mathrm{B}}\right) $$

When extended to the three factors (A, B, C), it becomes

$$ {PAF}_{ABC}=1-\left(1-{PAF}_{\mathrm{A}}\right)\ \mathrm{x}\ \left(1-{PAF}_{\mathrm{B}}\right)\ \mathrm{x}\ \left(1-{PAF}_{\mathrm{C}}\right) $$

Therefore, the summary PAFs simultaneously attributed to three risk factors in the current study was as describe below.

$$ {PAF}_{\mathrm{combined}}=1-\left(1-{PAF}_{\mathrm{physical}\ \mathrm{inactivity}}\right)\ \mathrm{x}\ \left(1-{PAF}_{\mathrm{overweight}}\right)\ \mathrm{x}\ \left(1-{PAF}_{\mathrm{alcohol}\ \mathrm{consumption}}\right) $$

As described elsewhere [16, 17], PAFs in adults were calculated for adult-onset cancers (i.e. CRC in this case), following exposures. We stratified PAFs by adult males and females or by ethnicity, based on availability of data.

To be robust, we also estimated PAFs by using the lower and upper boundaries of the 95% confidence interval (CI) for RR estimates and for prevalence data simultaneously. We, therefore, reported PAF with corresponding 95% CI.

### Assumptions

For calculation of PAF, it is necessary to make the assumptions; (i) the association between the exposure/consumption of the risk factor and the CRC was causal, (ii) the effect of this causal factor was independent of other causal factors, and (iii) a reasonable latent period was considered for the use of prevalence data.

## Results

**Fig. 1:**
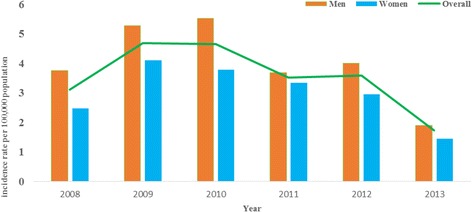
Colorectal cancer incidence rate per 100,000 population in Malaysia (2008–2013)

The temporal trend of CRC incidence from 2008 to 2013 was shown in Fig. 1. Overall, the mean incidence rate for CRC in Malaysia from 2008 to 2013 was 21.3 per 100,000 population, with the mean age of 61.6 years (±12.7) and the majority were men (56.6%). During this period, the registered CRC in Malaysia decreased for both men and women. For males, the rate decreased from 15.6 to 8.0%, while the rate decreased from 13.7 to 8.0% for women. Age-adjusted incidence rate of CRC was 1.33 times higher in men than women. When stratified by ethnicity, the highest incidence rate of 27.4 per 100,000 population was found amongst Chinese. Overall mortality rate for CRC was 9.8 per 100,000 populations. Age-adjusted mortality rate of CRC was about 1.42 times higher among males than females. A total of 369 CRC cases were reported in 2013 [5].

### Attributable fraction

The estimated PAFs for each of the three selected modifiable risk factors were provided in Table 2. The estimates are based on a key assumption that the factor of interest is causally related to CRC. PAFs for lack of physical activity were 10.1% (95% CI: 7–13%) in men and 11.6% (95% CI:6–17.4%) in women CRC cases. It was estimated that 20 (95% CI:14–26) men and 20 (95% CI:10–29) women CRC cases in 2013 would be avoided, if the risk factor (physical inactivity) was causally related to CRC and was prevented. Of them, 18 (95% CI: 11–27), 16 (95% CI: 9–23), 2 (95% CI: 1–4) and 4 (1–5) were Malays, Chinese, Indians and other ethnic groups, respectively.

**Table 2:** Estimation of combined population attributable fractions

Description	PAF	Multiplication (A x B x C)	Preventable cases (95% CI)^a^
Men (*n* = 201)			
Alcohol consumption	0.078 (0.038–0.129)	-	-
Overweight	0.045 (0.02–0.076)	-	-
Physical inactivity	0.101 (0.07–0.13)	-	-
Three combined factors		0.209 (0.123–0.3)	42 (25–60)
Women (*n* = 168)			
Alcohol consumption (A)	0.021 (0.001–0.055)	-	-
Overweight (B)	0.009 (0.02–0.076)	-	-
Physical inactivity (C)	0.116 (0.06–0.174)	-	-
Three combined factors		0.142 (0.08–0.279)	24 (13–47)

*PAF* population attributable fraction

^a^PAF_combined_ = 1 − (1 − PAF_physical inactivity_) × (1 − PAF_overweight_) × (1 − PAF_alcohol consumption_)

PAFs for overweight (25–29.9 body mass index, BMI) were 4.5% (95% CI: 2–7.6%) in men and 0.9% (95% CI: 0–2.8%) in women CRC cases. It was estimated that 9 (95% CI: 4–15) men and 1 (95% CI: 0–5) woman CRC cases would be avoided, if they could control their body weight through effective measures. This means 5(95% CI:1–6), 3 (95% CI:1–6), 1(95% CI: 0–1) and 1 (95% CI: 0–1) were CRC cases of Malays, Chinese, Indians and other ethnic groups, respectively.

For the alcohol consumption, PAFs for CRC in men and women were 7.8% (95% CI: 3.8–12.9%) and 2.1% (95% CI: 0–6%), respectively. It was estimated that 16 (95% CI: 8–26) men and 4 (95% CI: 0–9) women cases would be prevented from CRC in 2013, if they were not regular alcohol drinkers. Of them, 16 (95% CI: 7–25), 2 (95% CI: 1–3) and 2 (95% CI: 1–3) were Chinese, Indians and other ethnicity, respectively.

PAFs estimates for combined effect of the selected three factors were 12.7% (95% CI: 3–21.1%) in men and 8.6% (95% CI: 7.2–9.2%) in women CRC cases. It was estimated that 66 CRC cases (42 men, 24 women) who had all these three risk factors for the last 10 years would have been prevented in 2013, if they could control overweight, physical inactivity and alcohol consumption through effective preventive measures.

## Discussion

To our knowledge, this is the first study to estimate the PAF for three selected modifiable risk factors related to the CRC in Malaysia. The potential impact of preventive measures could be assessed by computing the PAF which represents the proportion of CRC cases in the Malaysia population that could be prevented, if exposure to a causal factor had not occurred.

The PAF compared with other countries are presented in Table 3. For physical inactivity, our findings showed a relatively lower PAF estimate of men than women, and this pattern is consistent with that in Korea [25] and Brazil [26]. A large scale survey with 19,145 Malaysians had shown that men residing in urban and rural settings were more physically active compared to their counterparts [27]. PAF for physical inactivity for both genders in the current study was greater than that in the Korean study [28] and the Brazil study [26]. Differences in prevalence proportion could explain this variation. For instance, 28.7% of men in Brazil [26] were considered physically inactive, while this was 35.3% in Malaysia [12]. Hence, even though RR estimates were extracted from the same meta-analysis [23], the PAF was larger in the current study than in the Brazil study [29]. Additionally, PAF is sensitive to risk estimation. For instance, physical inactivity in the Korean study [28] used RR of 1.01 for men and 1.11 for women CRC cases, while the present study used RR of 1.32 and 1.26 for men and women, respectively.

**Table 3:** Population attributable fraction by country

Risk factor of interest	Malaysia^a^	Korea^b^	UK^c^	Brazil^d^
Physical inactivity	M: 10.1% (95% CI: 7–13%)	M: 0.8%		M:8.4%^e^
	F: 11.6% (95% CI: 6–17.4%)	F: 0.9%		F:9%
Overweight	M:4.5% (95% CI: 2–7.6%)	M: 6.8%		M:15.8%
	F: 0.9% (95% CI: 0–2.8%)	F: 6.6%		F:7%
Alcohol drinks	M: 7.8% (95% CI: 3.8–12.9%)	M: 8.6%	M:15.5%	M: 2.7%
	F:2.1% (95% CI: 0–6%)	F: 4.2%	F:6.9%	F:0.25%

^a^present study

^b^[28]

^c^[25]

^d^[26]

^e^Recalculated value; F: female; M: male

The PAF for alcohol consumption for both genders in the current study was lower than that in the Korean [28] and the UK studies [25], but higher than in the Brazil study [26]. Variation of RR used in these studies and/or the differences in dose- risk relationship could also play a role. For instance, prevalence for alcohol consumption in the Malaysian national survey covered ‘current drinker’, while this was ‘dose-based drinker’ (3 U for men and 2 U for women) in the UK study [25].

Compared to that in Korea [28] and Brazil [26], a lower PAF for overweight in the CRC risk in Malaysia was observed. Differences in exposure prevalence could also explain this variation; for instance prevalence of overweight (25–30 BMI) in men was 41.02% in Brazil [26] and 29.7% in Malaysian [13]. It has also been reported that obesity was considered an important risk factor for many types of solid cancers, especially for CRC [8]. Although the exact mechanism of this association between CRC and overweight/increased BMI is still pending, there are possible explanations. For instance, the involvement of insulin and IGF-1 in colorectal carcinogenesis could have increased free IGF-I with concomitant changes of environment mitogenesis and anti-apoptosis in the cells favouring tumour formation [29]. Moreover, fatty tissue itself can also influence CRC risk as adipocytes and preadipocytes could promote proliferation of CRC cells [30]. In vivo and in vitro experiments showed that fatty acid synthase overexpression was associated with CRC phenotype [31].

Like studies elsewhere [25, 26, 28], the CRC risk in men associated with alcohol consumption in the current study was higher than women. This may be due to gender difference in practices that men had a relatively larger spectrum of alcohol intake and this allowed easier identification of an association, or that there may be hormone-related differences in alcohol metabolism [32]. Possible explanations for the lower PAF for alcohol consumption in Malaysia compared to other countries such as Korea and UK may be due to (i) a true low consumption of alcohol as it is not an acceptable cultural norm amongst Malays (i.e. the Islamic faith prohibits alcohol consumption), (ii) the use of self-reported alcohol consumption, and/or (iii) whether a large proportion of the Malaysian population had slow action of enzyme Aldehyde dehydrogenase (ALDH) responsible for detoxifying aldehyde in alcohol, which was found among the Korean population in Japan [33]. This latter point is supported by a finding that about 30% of East-Asian populations have the ALDH2*2 variant allele, and therefore usually avoid drinking alcohol, or drink lower quantities than other population groups [33].

The estimation of PAFs in this study has public health implications. The current study has suggested that 66 CRC cases (95% CI: 38–107) were attributable to the combined risk factors of physical inactivity, overweight and alcohol consumption; these are, of course, immediately modifiable factors. Hence, the estimates in the current study could serve as useful quantitative information to the national health policy makers for the development of CRC prevention and control strategies in Malaysia. A study in Malaysia showed that only small proportion could identify alcohol consumption (5.6%), overweight (2.8%) and physical inactivity (3.4%) as risk factors for CRC [34]. This imply that public education campaign for CRC prevention and control should focus more on the immediately modifiable risk factors for substantial reduction in the incidence of CRC and subsequent premature deaths.

### Study limitations

The national cancer registry covered the whole country, comprising Peninsular Malaysia, Sabah and Sarawak [7]. There might have been confirmed cancer cases yet to be notified to the cancer registry at the time of reporting. Hence, a possible underreporting of incidence in the NCR report is a concern. Moreover, participants in the primary studies included in meta-analyses might be confounded with comorbid diseases or possibility of interactions among risk factors, leading to uncertainties in RR estimates used in the current study. For estimation of combined PAFs, all risk factors were given the same weightage, duration of exposure to each risk factor was not taken into account and interaction between risk factors and genetics were not addressed. Hence, the combined PAFs in the current should be interpreted with cautions.

Additionally, due to limited prospective studies on association between risk factors and CRC in Malaysia, we used RR estimates extracted from the high quality published meta-analyses; a true representativeness to Malaysian population is a concern. Prevalence proportions were based on self-reported exposure. Misclassification bias related to underreporting of exposure or recall bias for recording (i.e. physical activity/alcohol consumption) might lead to an underestimation of the PAFs. Exposure prevalence applied in this study was aggregate data of the ethnic groups involved, although variation may exit among genders of each ethnic group. RR estimates were also obtained from multiple sources, which could be subjected to bias in PAF estimation. Prevalence data was obtained from a large scale national representative survey in 2006. Although we assumed 10 years of latent period, we could only use prevalence data with less than 10 years of latent period. We have assumed that the prevalence data for these risk factors among Malaysian population did not change overtime between 2003 and 2006. Moreover, this violation of an assumption is likely to be minimum as a true latent period for CRC is difficult to know.

Nevertheless, The RR estimates were derived from the pooled analysis, rather than a single study. Since the literature-derived RRs applied in the present analysis were already adjusted for most important confounders, it is less likely that remaining unmeasured confounders could have influenced the current findings considerably.

## Conclusions

Findings suggest that in Malaysia, approximately 18% of the CRC cases in the year 2013 would be preventable, if proper interventions to limit physical inactivity, overweight and alcohol consumptions were carried out. To reduce the incidence of CRC in Malaysia, scaling-up nationwide public health campaigns to educate the public to reduce alcohol intake, increase physical activity and control body weight are recommended. Future studies with other site-specific cancers and additional risk factors are needed.

## Abbreviations

ALDH: Aldehyde dehydrogenase
CI: Confidence interval
CRC: Colorectal cancer
IARC: International Agency for Research of Cancer
NCPR-CC: National Cancer Patient Registry
NHMS: National Health and Morbidity Survey
PAF: Population attributable fraction
RR: Relative risk
